# Supplementary material for: Ratanjot (Alkanna tinctoria L.) Root Extract, Rich in Antioxidants, Exhibits Strong Antimicrobial Activity against Foodborne Pathogens and Is a Potential Food Preservative
Source: Foods. 2024 Jul 17;13(14):2254. doi: 10.3390/foods13142254 (PMC11275321; doi:10.3390/foods13142254)
Supplement: Supplementary file 1 [file foods-13-02254-s001.zip › foods-3090811-supplementary.pdf]

Supplementary File

Supplementary Table S1. Formulation of chicken meatballs prepared with Ratanjot root extract powder (RRP)

Ingredients (%)	Control	T1	T2
Meat	72.0	71.0	69.5
Ice flakes	10.0	10.0	10.0
Refined vegetable oil	8.00	8.00	8.00
Salt	1.50	1.50	1.50
Condiment Mix*	3.50	3.50	3.50
Refined wheat flour	3.20	3.20	3.20
Dry spice mix	1.50	1.50	1.50
Sodium nitrite (ppm)	150	150	150
Polyphosphate	0.30	0.30	0.30
Ratanjot Root Extract Powder (RRP) %	--------	1.0	2.5

Condiments mix=Onion: Garlic (2:1)
